# Development of a quantitative ELISA for SARS-CoV-2 vaccine candidate, NDV-HXP-S, with CpG 1018® adjuvant

**DOI:** 10.1080/21645515.2024.2315709

**Published:** 2024-02-19

**Authors:** Marcus Estrada, Changcheng Zhu, Anan Bzami, Jessica A. White, Manjari Lal

**Affiliations:** Medical Devices and Health Technologies, PATH, Seattle, WA, USA

**Keywords:** ELISA, SARS-CoV-2, vaccine formulation, adjuvant, stability

## Abstract

NDV-HXP-S is a Newcastle disease virus (NDV) vectored vaccine candidate which expresses the S-antigen of severe acute respiratory syndrome coronavirus 2 (SARS-CoV-2). This vaccine candidate is under evaluation in human clinical studies with and without cytosine phosphate guanine (CpG) 1018® adjuvant. Existing potency methods for NDV-HXP-S do not allow for quantification of the S-antigen when the adjuvant is present. To support evaluation of NDV-HXP-S with CpG 1018® adjuvant, an inhibition enzyme-linked immunosorbent assay (ELISA) was developed to allow for quantification and stability assessments of the vaccine. A pilot 6-month stability study was conducted on NDV-HXP-S vaccine with and without CpG 1018® adjuvant under refrigerated conditions (2°C to 8°C) and accelerated stability testing conditions (40°C). The vaccine was mixed with and without CpG 1018® adjuvant in saline and maintained S-antigen content at 2°C to 8°C for the entire 6-month period. Additionally, a pilot controlled temperature chain (CTC) stability study was conducted at the completion of the 6-month study and demonstrated the possibility for this vaccine candidate to attain CTC stability labeling.

## Introduction

The pandemic caused by severe acute respiratory syndrome coronavirus 2 (SARS-CoV-2) has led to a surge in vaccine development. One inactivated vaccine candidate under development is based on a Newcastle disease virus (NDV) vector expressing the ectodomain of a novel, membrane-anchored, prefusion-stabilized Wuhan-Hu-1 spike protein construct from SARS-CoV-2, known as NDV-HXP-S.^1–3^ The NDV-HXP-S vaccine candidate leverages existing egg-based manufacturing capacity to produce an affordable SARS-CoV-2 vaccine. NDV-HXP-S is purified from allantoic fluid and then inactivated with beta propiolactone (b-P). Vaccines that are inactivated with b-P may have the advantages of an improved safety profile as well as the ability to be combined with an adjuvant for improved efficacy and dose-sparing compared to live vaccine candidates. This inactivated vaccine candidate displays a SARS-CoV-2 spike protein on the surface, where it is stabilized in its prefusion-conformation by the introduction of six proline mutations (HexaPro: HXP-S).^3^ The NDV-HXP-S vaccine candidate is being developed by manufacturers in Vietnam, Thailand, and Brazil, and is currently undergoing human clinical studies.^4,5^

Developed by Dynavax Technologies Corporation, CpG 1018® adjuvant is an oligonucleotide containing unmethylated cytosine and guanine bases connected by a phosphate group. This adjuvant is a potent stimulator of the innate immune system through the activation of toll-like receptor 9. It has been researched in both pre-clinical and clinical studies. Furthermore, CpG 1018® adjuvant is used in a licensed hepatitis B vaccine (HEPLISAV-B®) as well as in a number of SARS-CoV-2 vaccines that are fully approved or authorized for emergency use.^6^ CpG 1018® adjuvant is being evaluated for use in the NDV-HXP-S vaccine candidate.

Previously developed potency measurements for NDV-HXP-S involved a direct enzyme-linked immunosorbent assay (ELISA) to quantify and monitor the stability of the S-antigen present in the vaccine.^4^ However, the addition of CpG 1018® adjuvant to the direct ELISA showed decreased sensitivity to S-antigen content, possibly due to interference with the antigen coating of the ELISA plate. To evaluate the quantity and monitor stability of the S-antigen in NDV-HXP-S after the addition of CpG 1018® adjuvant, the development of an alternate potency method was required.

In this manuscript, we describe the development of an inhibition ELISA to quantify and monitor the stability of the S-antigen present in the NDV-HXP-S vaccine candidate when mixed with CpG 1018® adjuvant. This process included identifying appropriate antibodies for S-antigen detection, comparing methodology with previous potency measurements, and determining if the method effectively indicates stability. Once developed, this inhibition ELISA was used in a pilot 6-month study to evaluate the stability of NDV-HXP-S in the presence of CpG 1018® adjuvant. In addition, the inhibition ELISA was transferred to the vaccine manufacturers for method validation and application to both in-process drug substance samples and final drug products which contain both NDV-HXP-S and CpG 1018® adjuvant.

## Materials and methods

### NDV-HXP-S bulk, CpG 1018® adjuvant, and vaccine formulation

Bulk NDV-HXP-S vaccine lots were kindly provided by The Government Pharmaceutical Organization, Thailand (GPO) and Instituto Butantan, Brazil for use in the stability studies. Vaccine bulks were formulated to mimic final vaccine concentrations, vial fill volumes, and vial compositions being used in ongoing clinical studies.^4^ Bulk CpG 1018® adjuvant was kindly provided by Dynavax Technologies Corporation (Emeryville, California, United States).

CpG 1018® adjuvant content was measured according to methods provided by Dynavax Technologies. CpG 1018® adjuvant integrity was monitored using methods provided by Dynavax Technologies.

Bulk vaccine material was prepared with and without CpG 1018® adjuvant to achieve a final concentration of 2 µg/mL or 6 µg/mL S-antigen with and without 3 mg/mL CpG 1018® adjuvant in phosphate-buffered saline (PBS) buffer (Cytiva CAT# SH30028.03). Formulations were filled with 2.4 mL in 2 R glass vials prior to being placed at 2°C to 8°C or 40°C for stability studies.

### Direct ELISA

Direct ELISA potency testing was conducted as previously published on NDV-HXP-S samples mixed at 7.5 µg/mL S-antigen with 3 mg/mL CpG 1018® adjuvant in saline as well as NDV-HXP-S alone in saline at 25 µg/mL S-antigen.^4^ Both of these concentrations represent target doses of 3 µg S-antigen and 10 µg S-antigen in a 0.400 mL dose. The vaccine concentrations were further optimized for a typical 0.500 mL dose volume with target doses of 1 µg S-antigen and 3 µg S-antigen.

### Inhibition ELISA

Antibodies from both research and commercial sources were screened for use in the inhibition ELISA. These were kindly provided by Dr. Burton’s lab at The Scripps Research Institute (La Jolla, California, United States), or purchased from Sino Biological US Inc. and Leinco Technologies.^7^ Antibody sensitivity was evaluated across dilutions from 1 to 0.002 µg/mL both NDV-HXP-S coated on an ELISA plate and mixed in solution. Antibodies able to bind to the S-antigen present in the NDV-HXP-S vaccine candidate in solution were further down-selected based on the limit of detection to ensure the developed method could quantify expected vaccine doses.

After antibody selection, an inhibition ELISA was developed to quantify and monitor stability of the S-antigen present in NDV-HXP-S vaccine samples based on previously published methods.^8^ Blocking buffer, primary incubation time and temperature, and dilution scheme were optimized as part of method development. Briefly, NDV-HXP-S test samples and an NDV-HXP-S standard were diluted in assay buffer (PBS containing 0.05% Polysorbate 80 and 1% bovine serum albumin) to a starting concentration of approximately 5 µg/mL S-antigen and then further diluted 1.67-fold across a deep-well assay plate (VWR CAT# 76329–998) for a total of 11 dilutions. The primary antibody (Sino Biological selected for final use) was diluted to 0.054 µg/mL in assay buffer and added to the standard and test samples, which were incubated for 1 hour at room temperature (RT) with shaking at 500 revolutions per minute (RPM) using a digital microplate shaker (Thermo Scientific 88,880,023 Compact Digital Microplate Shaker) and then moved to 2°C to 8°C and held overnight. A high-binding ELISA plate (Corning CAT# 3690) was coated with 50 µl of NDV-HXP-S coating antigen at 1 µg/mL of S-antigen in PBS (Cytiva CAT# SH30028.03) and held overnight at 2°C to 8°C. After incubation, unbound primary antibody was quantified by transferring the supernatant from the deep-well plate to the coated ELISA plate and detected with a secondary antibody (Millipore CAT# AP503). Tetramethylbenzidine (TMB) liquid substrate (Sigma CAT# 0440) was added to the ELISA plate (50 µl per well) and incubated in the dark for 15 minutes. TMB reactions were stopped with 50 µl of 1N H2SO4 and the optical density (OD) values were read at 450 nm (SpectraMax® M2 plate reader from Molecular Devices equipped with SoftMax® Pro 7.1.2 software). The NDV-HXP-S standard was graphed to generate a standard curve using a four-parameter fit, and the test sample values were interpolated from the standard curve. Only the sample data points in the linear range of the standard were averaged to determine the final test sample value. Inhibition ELISA sensitivity to S-antigen in the presence of CpG 1018® adjuvant was evaluated by comparing the interpolated values of samples with and without CpG 1018® adjuvant. To determine if the developed method was stability indicating, NDV-HXP-S was exposed to heat-stress temperature conditions of 60°C for 15–20 minutes prior to ELISA testing.

The inhibition ELISA was evaluated according to the ICH guidelines by completing testing with two analysts on three separate days testing a panel of test samples. Intra and Inter assay CV, appropriate sensitivity for the target vaccine concentrations and then the ELISA technology was transferred to each of the vaccine manufacturing partners. Each of the manufacturing partners has validated the inhibition ELISA method for potency testing to support clinical studies and vaccine licensure.

### Stability studies

Samples for a 48-hour stability study were prepared at a final concentration of 6 µg/mL and 2 µg/mL S-antigen in saline with and without CpG 1018® adjuvant at 3 mg/mL. The bulk antigen was diluted to 12 µg/mL in PBS (Cytiva CAT# SH30028.03) and then diluted to the final S-antigen concentration with saline alone or saline mixed with CpG 1018® adjuvant, similar to the bedside mixing that was done for the clinical trial. Samples were then filled into 5 mL glass vials (2.4 mL for GPO bulk and 2.8 mL for Butantan), sealed with stoppers and crimps, and held at refrigerated temperature (2°C to 8°C) and RT (25°C) prior to testing. Duplicate vials were prepared for each formulation and two replicate vials were tested at each time point. All samples were held at 2°C to 8°C and 25°C prior to testing at 24 and 48 hours.

Six-month stability samples were prepared with NDV-HXP-S 6 µg/mL S-antigen in saline with and without 3 mg/mL of CpG 1018® adjuvant. The bulk antigen was diluted to 12 µg/mL in PBS and then diluted with saline (with and without CpG 1018® adjuvant) to the final concentration. Formulations were filled in sterile 2 R glass vials at 2.4 mL per vial. S-antigen and CpG 1018® adjuvant stability were monitored after storage at refrigerated temperature (2°C to 8°C) and accelerated testing (40°C) for the following timepoints using the methods described above: 1 day; 3 days; 1, 2, 4, and 6 weeks; 3 months; and 6 months. A frozen (−80°C) antigen control and a liquid antigen control (2°C to 8°C), both without CpG 1018® adjuvant, were tested throughout the stability study.

To evaluate the potential of the NDV-HXP-S vaccine to meet controlled temperature chain (CTC) criteria, vaccine stability was further evaluated after an excursion from the cold chain at the end of the 6-month study. After storage at 2°C to 8°C for 6 months, three vials were moved to 40°C and held for 3 days prior to ELISA testing. S-antigen content was monitored by inhibition ELISA after exposure to 40°C to satisfy CTC stability requirements at the end of the 6-month stability study with and without CpG 1018® adjuvant.

## Results

**Figure 1.**
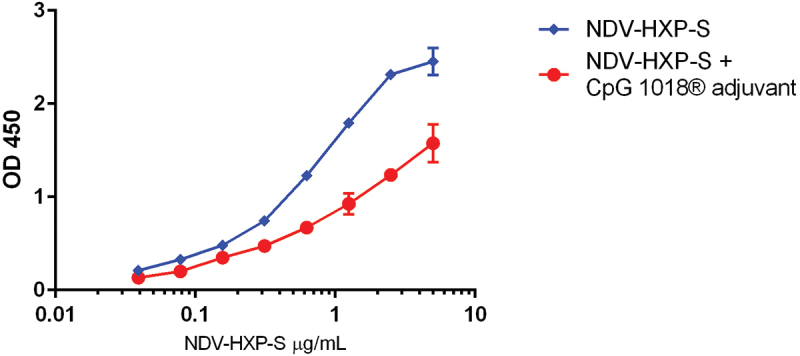
NDV-HXP-S S-antigen potency in the presence of CpG 1018® adjuvant by direct ELISA. NDV-HXP-S test samples containing 7.5 µg/mL S-antigen and 3 mg/mL CpG 1018® adjuvant (indicated by the red line) were prepared in saline and tested by direct ELISA compared to NDV-HXP-S samples prepared at 25 µg/mL S-antigen in saline without CpG 1018® adjuvant (indicated by the blue line).

Initial testing of NDV-HXP-S in the presence of CpG 1018® adjuvant was conducted using the previously published direct potency ELISA using CR-3022 antibody for S-antigen quantification.^4,9^ As seen in Figure 1, the sensitivity of the ELISA to S-antigen content decreased and the method was no longer able to reliably quantify the dose of S-antigen in the presence of CpG 1018® adjuvant planned for the clinical studies. The NDV-HXP-S test sample without CpG 1018® adjuvant shown in blue below generates a full sigmodal curve with a top and bottom plateau however samples containing the CpG 1018® adjuvant shown in red do not produce a sigmodal curve or a top or bottom plateau. Samples of NDV-HXP-S with CpG 1018® adjuvant are not parallel to the reference standard and values cannot be interpolated to provide the S-antigen content. This is likely due to interference from CpG 1018® adjuvant with the binding kinetics to the ELISA plate in the direct ELISA. Attempts to prepare the test sample prior to ELISA testing by filtration to remove the adjuvant were unsuccessful. Since the direct ELISA could not be used to quantify the S-antigen content in the NDV-HXP-S vaccine when CpG 1018® adjuvant was present, an alternate potency method was established. Continued use of the direct ELISA would require use of more than one standard for interpolation of samples with and without CpG 1018® adjuvant, essentially requiring two separate methods. Due to the lack of sensitivity and the need for multiple methods the direct ELISA was not pursued further.

**Figure 2.**
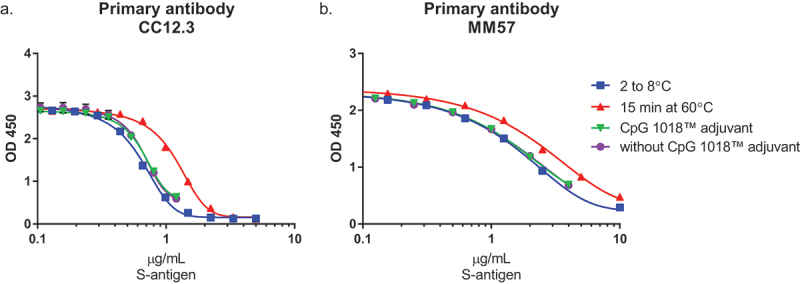
S-antigen inhibition ELISA development. Primary antibodies were screened for their ability to quantify S-antigen in the presence of CpG 1018® adjuvant and after damage to NDV-HXP-S by exposure to heat 15–20 min at 60°C, with (a) showing the results for primary antibody CC12.3 and (b) the results for primary antibody MM57.

Multiple antibodies were screened for potential use in the inhibition ELISA from both academic and commercial sources. Both CC12.3 (Scripps Research Institute) and MM57 were identified for use as primary antibodies due to their sensitivity to antigen content, recognition of a neutralizing epitope, and ability to detect changes in the S-antigen after exposure to heat (Figure 2). While the ELISA curves for both antibodies have slightly different shapes, the limits of detection are relatively similar, and the inhibition assay is conducted as a relative potency measure with a reference standard included on each assay plate (see the blue lines in Figure 2).

**Figure 3.**
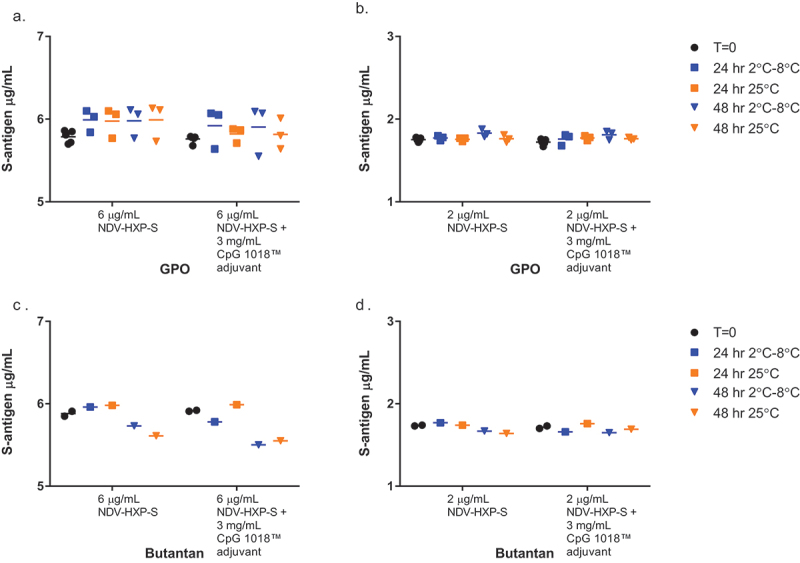
Pilot S-antigen stability. Stability of NDV-HXP-S at 2°C to 8°C and 25°C for 48 hours. (a) GPO NDV-HXP-S at 6 µg/mL with and without CpG 1018® adjuvant at 3 mg/mL; (b) GPO NDV-HXP-S at 2 µg/mL with and without CpG 1018® adjuvant at 3 mg/mL; (c) Butantan NDV-HXP-S at 6 µg/mL with and without CpG 1018® adjuvant at 3 mg/mL; (d) Butantan NDV-HXP-S at 2 µg/mL with and without CpG 1018® adjuvant at 3 mg/mL.

Antibody CC12.3 was further evaluated in a 48-hour stability study with and without CpG 1018® adjuvant at both relevant clinical dose levels of the S-antigen (Figure 3). Vaccine mixing and testing were conducted to mimic procedures used in both toxicology and human Phase I clinical studies. Stability of the NDV-HXP-S vaccine candidate at 2 µg/mL and 6 µg/mL S-antigen with and without 3 mg/mL CpG 1018® adjuvant were evaluated using the inhibition ELISA after being held for 48 hours at 2°C to 8°C and 25°C. NDV-HXP-S vaccine stability was based on maintaining the S-antigen content by ELISA within 30% of the expected S-antigen content   for all stability tests. The changes observed in S-antigen content over the 48-hour testing period, as shown in Figure 3, are within the variability of the ELISA and are not considered significant differences. Due to limited test materials only one replicate was tested for the Butantan samples however the stability trend was consistent between manufacturers.

Parameters of the developed inhibition ELISA including sensitivity, specificity, and reproducibility are shown in Table 1 and were evaluated according to International Conference on Harmonization (ICH) guidelines.

**Table 1.** Inhibition ELISA parameters.

Parameter	Specification
Specificity	SARS-CoV-2 S-antigen
Antibody	MM57
Reproducibility (%CV)	3%
Intra-assay (%CV)	6.5%
Inter-assay (%CV)	9.0%
Background (OD 450 ± SD)	0.071 ± 0.01

**Figure 4.**
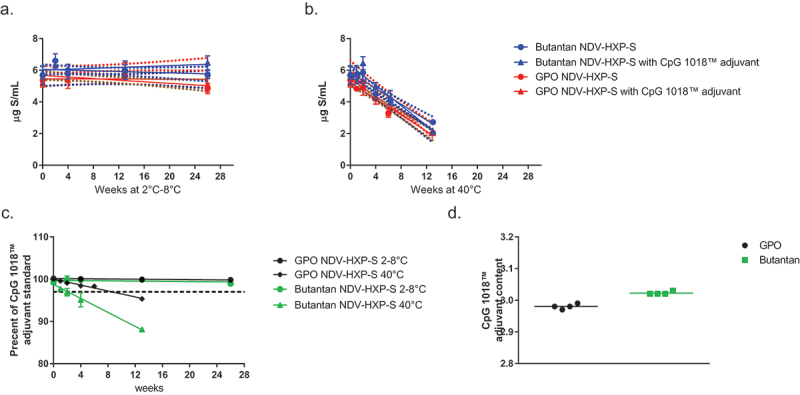
6-month stability of NDV-HXP-S with and without CpG 1018® adjuvant. NDV-HXP-S vaccine was prepared at 6 µg/mL S-antigen with and without 3 mg/mL CpG 1018® adjuvant in saline and held at 2°C to 8°C and 40°C. Samples were removed from temperature and tested for S-antigen content and stability by inhibition ELISA at selected timepoints. (a) NDV-HXP-S stability at 2°C to 8°C; (b) NDV-HXP-S stability at 40°C; (c) CpG 1018® adjuvant stability (integrity) at 2°C to 8°C and 40°C shown as a percentage of a CpG 1018® adjuvant standard tested at each time point; and (d) CpG 1018® adjuvant content at the beginning of the stability study. Methods provided by Dynavax technologies.

Based on the 48-hour stability study, a 6-month stability study was conducted to further evaluate stability of the NDV-HXP-S vaccine candidate using bulk vaccine material from both vaccine manufacturers. Samples were prepared containing 6 µg/mL S-antigen with and without 3 mg/mL CpG 1018® adjuvant with saline and held at 2°C to 8°C and 40°C prior to testing. Stability was evaluated based on the ability of the formulation to maintain the S-antigen content between 4 µg/mL and 8 µg/mL. S-antigen content was tested using the inhibition ELISA, and CpG 1018® adjuvant integrity was tested using methods provided by Dynavax. Stability of the S-antigen and CpG 1018® adjuvant is shown in Figure 4. The S-antigen content was maintained for the entire 6-month period for all samples held at 2°C to 8°C, regardless of the presence of CpG 1018® adjuvant. None of the samples held at 40°C were able to maintain antigen and CpG 1018® adjuvant stability for the duration of the study.

**Figure 5.**
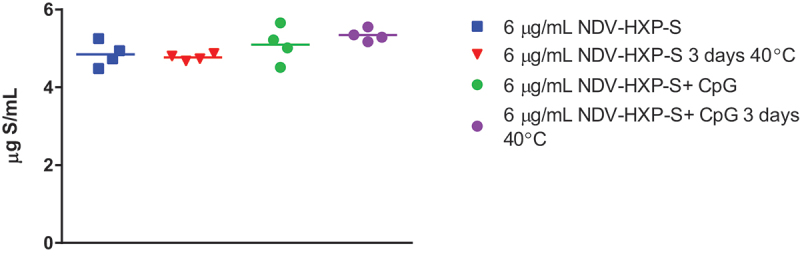
NDV-HXP-S controlled temperature chain stability. NDV-HXP-S vaccine was prepared at 6 µg/mL S-antigen with and without 3 mg/mL CpG 1018® adjuvant in saline and held at 2°C to 8°C for 6 months. After 6 months samples were moved to 40°C and held for 3 days prior to testing for S-antigen content by inhibition ELISA.

To understand the stability of the NDV-HXP-S vaccine outside of cold-chain storage, a CTC study was conducted at the end of the 6-month stability study with GPO material. After holding vials at 2 to 8°C for 6 months, three vials were moved to 40°C and held for 3 days prior to testing by inhibition ELISA. No difference in S-antigen stability was observed after 3 days at 40°C with or without CpG 1018® adjuvant present, Figure 5. The adjuvant stability was not evaluated in this study.

## Discussion

There is previous reported work on the development of a SARS-CoV-2 vaccine candidate referred to as NDV-HXP-S which has been shown to be manufactured at a low cost in embryonated chicken eggs, similar to the manufacturing process prevalent for influenza virus vaccines.^1,2^ This vaccine candidate is administered intramuscularly and has been tested with various adjuvants for dose sparing effects.^2^

The work presented here focuses on development of a sensitive, stability-indicating ELISA specific for the S-antigen content, which allows for quantification of NDV-HXP-S in the presence of CpG 1018® adjuvant to support the development of this vaccine candidate.

The selection of the antigen (3 µg/mL and 6 µg/mL) and the adjuvant dose (3 mg/mL) presented in this work was based on the dosing scheme followed in the clinical study.^4,5^ Although a direct ELISA has been shown to be successful for detection and quantification of S-antigen in NDV-HXP-S (vaccine alone preparations), it loses significant sensitivity in the presence of CpG 1018® adjuvant, as shown in our data. It is possible that the adjuvant is interfering with either immobilization of antigen on the ELISA plate through nonspecific binding and/or interfering with the binding of the detection antibody to the antigen in the direct ELISA approach. This test method would potentially pose a challenge for monitoring vaccine stability as we developed an adjuvant co-formulated vaccine product.

In the reported work, an inhibition ELISA was developed for quantification of the S-antigen in the presence of CpG 1018® adjuvant, based on previously published methods with a different vaccine candidate.^8^ Antibodies were selected based on specificity for the S-antigen expressed in the NDV-HXP-S vaccine candidate in the presence of CpG 1018® adjuvant; sensitivity at a relevant antigen dose; sensitivity to damage or changes in the S-antigen; recognition of a neutralizing epitope; and supply availability for future use by each of the manufacturing partners. Antibodies were screened for sensitivity to NDV-HXP-S and selected based on a relevant dose concentration. Antibodies that recognized a neutralizing epitope were prioritized to allow for potential bridging of *in vitro* potency values to relevant clinical outcomes. While initial method development identified both CC12.3 and MM57 as appropriate primary antibodies for use in the inhibition ELISA, in order to ensure long-term antibody supply to each of the manufacturing partners, a commercially available mouse monoclonal antibody specific for a neutralizing epitope on the receptor-binding domain (RBD) antigen was selected for future use in this method.

The inhibition ELISA provides the benefit of no interference from either the adjuvant or other impurities which may be present in the test samples. The interference observed with quantification of the S-antigen due to the presence of the CpG 1018® adjuvant is an interesting observation that requires further study. This allows for use of the inhibition ELISA to evaluate in-process drug substance samples allowing for more targeted process optimization and a consistent potency measure throughout the vaccine manufacturing process. The developed method is sensitive to the relevant S-antigen dose range being evaluated in clinical studies and was demonstrated to be stability indicating.

This method has been transferred to three manufacturing partners and can be used to support in-process testing, drug substance testing, vaccine release and stability studies. In order to support further development of this vaccine candidate, we describe initial pilot studies to understand baseline liquid stability of this unformulated vaccine candidate NDV-HXP-S with and without CpG 1018® adjuvant.

While the NDV-HXP-S vaccine candidate including the CpG 1018® adjuvant demonstrated stability for both antigen and the adjuvant for a period of 6 months at 2°C to 8°C, additional studies are needed to demonstrate the shelf-life stability of the vaccine product, appropriate product format to improve thermostability and the need for a preservative in a multidose presentation to reduce vaccine wastage and lower cost of the product. In this work, a pilot CTC study was conducted to understand the stability of the vaccine to intermittent elevated temperature exposure. WHO’s controlled temperature chain (CTC) program is a specific approach to vaccine management that allows vaccines to be kept at temperatures above the long-term storage condition for a limited period of time. Current WHO program conditions for CTC include a single exposure just prior to administration, tolerating ambient temperatures of at least 40°C for a limited duration of at least 3 days.

The pilot CTC study demonstrated the potential stability of the NDV-HXP-S vaccine candidate and future work with optimized vaccine formulations should be evaluated by vaccine manufacturers.

## Abbreviations

b-P: beta propiolactone
CpG: cytosine phosphate guanonine
CTC: controlled temperature chain
ELISA: enzyme-linked immunosorbent assay
GPO: Government Pharmaceutical Organization, Thailand
ICH: International Conference on Harmonization
NDV: Newcastle disease virus
OD: optical density
PBS: phosphate-buffered saline
RBD: receptor binding domain
RPM: revolutions per minute
RT: room temperature
SARS-CoV-2: severe acute respiratory syndrome coronavirus 2
TMB: tetramethylbenzidine
